# Features of Rural Communities in Latin America and Sub-Saharan Africa That Influence Well-Being of Older Persons

**DOI:** 10.1093/geroni/igab046.1622

**Published:** 2021-12-17

**Authors:** Andrew Banda, Norah Keating, Jaco Hoffman, Jose Parodi, Nereide Curreri

**Affiliations:** 1 University of Zambia, Lusaka, Lusaka, Zambia; 2 Swansea University, Swansea, Wales, United Kingdom; 3 North-West University, Vanderbijlpark, Gauteng, South Africa; 4 San Martin de Porres University, Lima, Lima, Peru; 5 North-West University, South Africa, Vanderbijlpark, North-West, South Africa

## Abstract

In their recent volume, Critical Rural Gerontology, Skinner et al (2021) challenge us to set aside unidimensional notions of rural communities as bypassed vs very supportive; and to identify the elements of rurality that empower or exclude older people and how these differ across cultures and settings. Covid-19 has highlighted the need for safe and inclusive communities. Given that LMIC will be home to the majority of older adults (Gonzales et al. 2015), we undertook a scoping review of features of rural communities that influence wellbeing of older people in countries across Latin America and Sub-Saharan Africa. The review included literature in English, French, Spanish and Portuguese, using search engines MEDLINE, CINAHL Complete, PsycInfo, SocINDEX, SciELO, AJOL (Africa Journals Online), LILACS, Redalyc, LatinIndex and Clacso. Findings illustrate diversity in how community features including remoteness, infrastructure and belonging influence material, social and subjective wellbeing of older residents.

